# *Pneumocystis jirovecii* colonization in chronic pulmonary disease

**DOI:** 10.1051/parasite/2011182121

**Published:** 2011-05-15

**Authors:** S. Gutiérrez, N. Respaldiza, E. Campano, M.T. Martínez-Risquez, E.J. Calderón, C. De La Horra

**Affiliations:** 1 Instituto de Biomedicina de Sevilla, Hospital Universitario Virgen del Rocío/CSIC/Universidad de Sevilla Seville Spain; 2 Servicio de Medicina Interna Hospital Universitario Virgen del Rocío Seville Spain; 3 CIBER en Epidemiología y Salud Pública, Hospital Universitario Virgen del Rocío Seville Spain

**Keywords:** *Pneumocystis jirovecii*, colonization, chronic pulmonary disease, *Pneumocystis jirovecii*, colonisation, broncho-pneumopathie chronique

## Abstract

*Pneumocystis jirovecii* causes pneumonia in immunosuppressed individuals. However, it has been reported the detection of low levels of *Pneumocystis* DNA in patients without signs and symptoms of pneumonia, which likely represents colonization. Several studies performed in animals models and in humans have demonstrated that *Pneumocystis* induces a local and a systemic response in the host. Since *P. jirovecii* colonization has been found in patients with chronic pulmonary diseases it has been suggested that *P. jirovecii* may play a role in the physiopathology and progression of those diseases. In this report we revise *P. jirovecii* colonization in different chronic pulmonary diseases such us, chronic obstructive pulmonary disease, interstitial lung diseases, cystic fibrosis and lung cancer.

## Abstract (fr)

*Pneumocystis jirovecii* est un agent de pneumonie chez les sujets immunodéprimés. Des faibles taux d’ADN de *Pneumocystis* peuvent cependant être détectés chez des patients sans pneumonie ; ils correspondraient à des situations de colonisation. Plusieurs études développées chez l’animal et chez l’homme ont montré que la colonisation par *Pneumocystis* peut induire chez l’hôte des réponses locale et systémique. Comme la colonisation peut être observée chez des patients atteints d’affections pulmonaires chroniques, il a été suggéré que *P. jirovecii* pourrait jouer un rôle dans la physiopathologie et l’aggravation de ces maladies. Dans cette revue, nous examinons la colonisation par *P. jirovecii* chez des patients atteints de maladies pulmonaires chroniques comme la bronchopneumopathie chronique obstructive, les fibroses pulmonaires interstitielles, la fibrose kystique et le cancer du poumon.

## Introduction

*Pneumocystis jirovecii* (human-derived *Pneumocystis*) is an atypical opportunistic fungus with lung tropism and worldwide distribution that causes pneumonia in immunosuppressed individuals (Calderon *et al.*, Calderon *et al.*, 2010). Although non-immunosuppressed patients rarely develop *Pneumocystis pneumonia* (PcP) (Cano *et al.*, 1993), it has been demonstrated that in respiratory samples of some subjects without signs and symptoms of pneumonia, can be detected low levels of *Pneumocystis* DNA that likely represent colonization (Wissmann *et al.*, 2010). Other terms that have been used include “carriage”, “subclinical infection” and “asymptomatic infection” (Peterson *et al.*, 2005).

The first evidence reported of *P. jirovecii* colonization in patients with chronic bronchial diseases without immunosuppression was observed by using immunofluorescence with monoclonal antibodies and standard stains. It was found that 10 % of patients was colonized (Calderón *et al.*, 1996). Current detection of *Pneumocystis* colonization has been greatly facilitated by the development of sensitive molecular techniques, such as polymerase chain reaction (PCR) (Calderón *et al.*, 2010). The rate of *P. jirovecii* colonization reported in chronic pulmonary diseases is diverse as a result of the used different sampling or detection procedures and the area where the study was performed (see Morris, Wei, *et al.*, 2008b for review), since *P. jirovecii* infection is apparently linked with environmental factors and probably there are low and high-risk geographical areas (Varela *et al.*, 2004).

It has been reported that *Pneumocystis* induce in animals models alveolar macrophage activation, elevation of proinflammatory interleukins, changes in lung surfactant, type I pneumocyte degenerative changes, type II pneumocyte reparatory hypertrophy, alterations in alveolus-capillary permeability, diffuse alveolar damage and lung fibrosis (Prévost *et al.*, 1998; Rice *et al.*, 1993; ThomaS *et al.*, 2007). In addition, a study using a culture system revealed that *Pneumocystis* major surface glycoprotein induces interleukin (IL)-8 and monocyte chemoattractant protein-1 release from a human alveolar epithelial cell line (Benfield *et al.*, 1999). Besides, it has been recently demonstrated that colonized patients with chronic obstructive pulmonary disease (COPD) have higher systemic proinflammatory cytokine levels than do noncolonized patients (Calderón *et al.*, 2007). These data suggest that *Pneumocystis* could play a role in the progression of chronic pulmonary diseases.

On the other hand, colonized patients can act as a reservoir and source of infection for susceptible subjects. In fact, the first molecular evidence that transmission of *P. jirovecii* from colonized immunocompetent carriers to susceptible subjects may occur has been recently provided (Rivero *et al.*, 2008). What’s is more, colonized individuals could represent a reservoir to *P. jirovecii* strains with dihydropteroate synthase (DHPS) gene mutations, potentially related with sulfadrugs resistance (Stein *et al.*, 2004). In this sense, the finding of *Pneumocystis* DHPS gene mutations in two colonized patients with chronic bronchitis, who were not previously exposed to sulfa drugs (Calderón *et al.*, 2004), suggests either possible occurrence of spontaneous mutations or, most probably, transmission of mutant strains from HIV-infected patients (frequently submitted to sulfa prophylaxis).

In this report we revise our current knowledge about *P. jirovecii* colonization in four major chronic lung diseases: COPD, interstitial lung diseases (ILDs), cystic fibrosis (CF) and lung cancer.

## Chronic Obstructive Pulmonary Disease

COPD is a major cause of illness, death, and the use of health care resources globally. The disease causes approximately 2.75 million deaths annually, and the number is projected to increase. At present, it is the 4th most common cause of death among adults, and it is estimated to rank 3rd by 2020 (Chapman *et al.*, 2006; Lopez *et al.*, 2006). COPD is a slowly progressive condition characterized by airflow limitation that is not fully reversible. The airflow limitation is associated with a chronic inflammatory response in both airways and lung parenchyma (Macnee, 2005). Smoking is considered to be the major cause of COPD, but only a small portion of all smokers develops the disease. It has been hypothesized that cofactors, as *P. jirovecii* colonization, may play a role in COPD development (Morris, *et al.*, 2008a). Additionally, smoking would be a risk factor for *P. jirovecii* colonization in both HIV-infected individuals (Morris *et al.*, 2004a) and subjects with chronic pulmonary diseases (Vidal *et al.*, 2006). Actually, cigarette smoking results in impaired mucociliary clearance, which could facilitate *P. jirovecii* colonization. In short, *Pneumocystis* lower respiratory tract colonization, together with smoking, could be a cofactor that increase or maintain inflammatory response, accelerating COPD progression. The rate of *P. jirovecii* colonization reported among patients with COPD is usually high. It shows however significant differences (16 to 55 %) that suggest major geographical disparities in *P. jirovecii* epidemiology (Calderón *et al.*, 2007; Nevez *et al.*, 2006). Interestingly, among the COPD patients from a same geographical area *P. jirovecii* colonization is more prevalent in those with severe COPD (Morris et al., 2004b)

β-glucans are major structural components of cyst wall of *Pneumocystis* (Lebrón *et al.*, 2003). β-glucans potently activate alveolar macrophages to release pro-inflammatory cytokines and chemokines through nuclear factor-κB (NF-κB) utilizing cellular receptors and signalling pathways distinct from bacterial lipopolysaccharide. β-glucans induce both cytokine generation and associated neutrophilic lung inflammation. In addition to *Pneumocystis* clearance, macrophages also strongly initiate inflammatory responses in the lung following interactions with β-glucan components of the organism, including secretion of tumor necrosis factor-α (TNF-α), IL-6, IL-8, macrophage inflammatory protein-2, eicosanoid metabolites, and reactive oxidant species (Lebrón *et al.*, 2003). These exuberant host inflammatory responses, though necessary for clearance of infection, further promote lung injury resulting in airspace inflammation and excessive morbidity. The pulmonary inflammatory response against *Pneumocystis* resembles that associated with COPD and could therefore contribute to the pathogenesis of airway and parenchyma damage in subjects with this disease (Morris *et al.*, 2008a).

An interesting study showed that *Pneumocystis* colonization correlates with degree of airway obstruction independent of smoking history (Morris et al., 2004b). This study did not prove causality, but it strongly suggests that *Pneumocystis* colonization affects COPD progression. In this sense, patients with COPD colonized by *P. jirovecii* were found to develop a higher systemic inflammatory response (increased levels of several circulating cytokines and acute-phase reactants including IL-8, IL-6 and TNF-α) than do noncolonized patients (Calderón *et al.*, 2007). In COPD patients, systemic inflammatory response has been linked to weight loss, muscle wasting and accelerate decrease in lung function (Gan *et al.*, 2004). As a cause of systemic inflammatory response, *Pneumocystis* colonization could therefore play a role in the pathophysiology of COPD.

## Interstitial Lung Diseases

ILDs are a heterogeneous group of disorders of the lower respiratory tract in which injury to the lung parenchyma leads to a cascade of inflammatory and immune processes that cause lung fibrosis. The exact mechanism by which connective tissue proliferation occurs in some cases is unknown, although infectious agents have been implicated in the pathogenesis of ILDs (Egan *et al.*, 1997, du Bois *et al.*, 2003). In this way, it has been suggested that pulmonary inflammation, together with the adaptive immune response triggered by alveolar injury caused by *Pneumocystis*, could play a role in the pathogenesis of ILDs and in the progression of the disease to fibrosis. The rate of *P. jirovecii* colonization in patients with ILDs ranged from 30 to 34 % (Vidal *et al.*, 2006; Martín-Juan *et al.*, 2008; Shimizu *et al.*, 2009) and it is similar among different kind of ILDs (Martín-Juan *et al.*, 2008) (Table I).

**Table I.** Prevalence of *Pneumocystis* colonization in individuals with interstitial lung diseases from Andalucía (Spain).

Interstitial lung diseases	Patients studied (n)	Patients with *P*. *jirovecii* colonization detected by molecular techniques (%)	Patients with *P. jirovecii* colonization detected by conventional and immunofluorescence staining (%)
Idiopathic pulmonary fibrosis	69	22 (31.8 %)	4 (5.7 %)
Fibrosing lung disease associated with connective tissue disease	38	13 (34.2 %)	2 (5.2 %)
Sarcoidosis	35	10 (28.5 %)	2 (5.7 %)
Cryptogenic organizing pneumonia	31	9 (29 %)	1 (3.2 %)
Diffuse alveolar haemorrhage	20	8 (40 %)	2 (10 %)

Modified from Martín-Juan *et al.*, 2008.

Interestingly, in a large study carried out in 240 patients with ILDS (Martín-Juan *et al.*, 2008), patients with ILDs who were carriers of *P. jirovecii* showed a significant higher peripheral blood total leukocyte and eosinophil levels compare to non-carriers. Also, the differential cell counts in the bronchoalveolar lavage fluid revealed a low CD4 ⁄ CD8 ratio in carrier patients, mainly because of an increased CD8+ T-cell count, although the differences did not reach statistical significance. This finding suggests that *Pneumocystis* colonization may be related to an exacerbation of these diseases. Further studies are needed in order to confirm the clinical significance of these findings.

## Cystic Fibrosis

CF is the most common serious hereditary disorder in the Caucasian populations. It is caused by several mutations in the CF transmembrane conductance regulator gene that map on the large arm of chromosome 7 (7q31.2) and encodes a chloride channel involved in electrolytic exchanges through the plasma membrane, leading to a defect in the mucociliary clearance and a thickness of the bronchial mucus (Rowe *et al.*, 2005). Several organs are affected in this disease and pulmonary damage is the most serious clinical problem. An important factor that contributes to the development of pulmonary disease in patients with CF is persistent infection by opportunistic pathogens, mainly *Pseudomonas aeruginosa*, *Staphylococcus aureus*, *Haemophilus influenza* and *Burkholderia cepacia* (Rowe *et al.*, 2005).

Recent researches indicate that a wide range of fungi can colonize the respiratory tract of patients with CF. *Aspergillus fumigatus* is the main fungus involved in fungal-related CF complication, however it is not the fungal infection itself which is mainly responsible for respiratory failure, instead the fungus grows in the upper respiratory way of CF patients, where it provokes repeated immune stimulation, such mechanism leads to Allergic bronchopulmonary aspergillosis (ABPA), a chronic hypersensitivity condition that can lead to morbidity and ultimately to respiratory failure (Antunes *et al.*, 2010).

Only a few published studies carried out in Europe have evaluated the prevalence of *Pneumocystis* colonization in patients with CF, reporting ranges from 1.3 % to 21.6 % (Table II) (Sing *et al.*, 2001; Respaldiza *et al.*, 2005; Nevez *et al.*, 2010). These differences in colonization rates could be related with differences in use of chemoprophylaxis with sulfa-drugs to prevent bacterial infections among the CF patients (Sing *et al.*, 2001; Respaldiza *et al.*, 2005; Gal *et al.*, 2010). However, they could also be due to differences in population density, and in HIV infection or PcP incidence (Gal *et al.*, 2010). On the other hand, several studies have shown seasonal changes of PcP incidence that seem to be associated with climate factors (Lubis *et al.*, 2003; Varela *et al.*, 2004; Sing *et al.*, 2009). In this sense, the higher *P. jirovecii* colonization rates observed in patients with CF from Southern Europe than in patients from Northern Europe could be related with climate factors (Sing *et al.*, 2001; Respaldiza *et al.*, 2005; Gal *et al.*, 2010).

**Table II.** Prevalence of *Pneumocystis jirovecii* colonization detected by using molecular methods among patients with cystic fibrosis.

Country	Number of patients	Prevalence of *Pneumocystis* colonization (%)	Median age (years)	Chemoprophylaxis with co-trimoxazole (%)	Authors, year
Germany	95	7.4 %	23.2	No	Sing et al., 2001
Spain	88	21.6 %	15.8	2.3 %	Respaldiza et al., 2005
France	76	1.3 %	18.5	39.5 %	Gal et al., 2010

*Pneumocystis* colonization does not appear to be a risk factor for subsequent PcP in CF patients, probably because the immune status of these patients is usually enough to control the colonization to pneumonia shift (Sing *et al.*, 2001; Respaldiza *et al.*, 2005; Montes-Cano *et al.*, 2007). However, even without deep immunosuppression, in chronic lung conditions *Pneumocystis* colonization could provoke mild or moderate respiratory illness, with the pathogen acting as a co-morbidity factor that may stimulate pulmonary inflammation and may play a pathogenic role (Calderón *et al.*, 2007; Morris *et al.*, Morris et al., 2008b). Thus, it would be hypothesized that *P. jirovecii* colonization provokes airflow obstruction and inflammatory response in CF patients. However, to our knowledge, no evidence supporting this hypothesis was provided till now. In other words, it has not yet been established whether *P. jirovecii* colonization could have a role in worsening the natural course of CF.

On the other hand, our knowledge of how *P. jirovecii* interact with pathogenic bacteria in the respiratory tract of patients with CF is completely unknown but this interaction could have some impact in the pathophysiology of CF. In this sense, a potential antagonistic relation among *P. jirovecii* colonization and bacterial microbiota has been recently reported in the respiratory tract of patients with idiopathic interstitial pneumonia (Friaza *et al.*, 2010).

## Lung Cancer

Tobacco smoking is the most important but not the only risk factor in lung carcinoma. The role of infectious agents in the development of human tumours was not demonstrated until recently (Kuper *et al.*, 2000). Actually, it is estimated that 15 % of all human cancers worldwide have an infectious origin (Pisani *et al.*, 1997). Recently, it has been reported the first evidence of an association between small cell lung carcinoma and the presence of *P. jirovecii* colonization. In this study, in all the examined samples of small cell lung carcinoma *P. jirovecii* was detected (it was not found in metastases) (de la Horra *et al.*, 2004). While the nature of this association is unclear, it nevertheless constitutes an important finding and open a new field for research since it is unknown either the *P. jirovecii* colonization is specifically facilitated by this tumour or induces the development of this type of neoplasm in combination with other factors.

## Conclusions

There is a high prevalence of *P. jirovecii* colonization in patients with chronic pulmonary diseases (Calderón, 2009). Data from different studies suggest a potential role of *P. jirovecii* in the pathophysiology of chronic pulmonary diseases through inducing inflammatory changes with chronic lung injury, but also interacting with other agents as tobacco or pathogenic bacteria (Calderón *et al.*, 2007; Morris *et al.*, 2008a; Christensen *et al.*, 2008; Friaza *et al.*, 2010). Understanding the role of *P. jirovecii* colonization in patients with chronic lung diseases may help us identify a novel, treatable pathogenic agent.

On the other hand, *P. jirovecii* colonization in patients with chronic pulmonary diseases could represent a problem for public health since colonized patients could act as a major reservoir and source of infection for susceptible subjects (Calderón, 2009; Wissmann *et al.*, 2010). In the future, advances as real time PCR could enhanced the sensitivity of *Pneumocystis* DNA detection and to make research on *Pneumocystis* colonization epidemiology much easier. In this way, the role of non invasive marker serum as β-glucan levels in colonized patients should be explored (Krajicek *et al.*, 2008).

Further investigation is need to better define the epidemiology and the role of *P. jirovecii* colonization in the natural course of chronic pulmonary diseases and to determine the exact mechanism of its effects.
